# Influence of screw channel angulation on reverse torque value and fracture resistance in monolithic zirconia restorations after thermomechanical cycling: an in-vitro study

**DOI:** 10.1186/s12903-024-04171-3

**Published:** 2024-03-26

**Authors:** Amir Alireza Khaledi, Shouka Shalileh, Maryam Hejazi, Rashin Giti

**Affiliations:** Department of Prosthodontics, Faculty of Dentistry, Shiraz University of Medical Sciences, Shiraz, Fars Iran

**Keywords:** Angulation, Dental implants, Dental abutments, Dental restoration failure, Fracture strength, Torque, Mechanical fatigue, Thermal cycling

## Abstract

**Background:**

While the concept of angled screw channels has gained popularity, there remains a scarcity of research concerning the torque loss and fracture strength of monolithic zirconia restorations with various screw channel angulations when exposed to thermomechanical cycling. This in-vitro study aimed to evaluate the reverse torque value and fracture resistance of one-piece screw-retained hybrid monolithic zirconia restorations with angulated screw channels after thermomechanical cycling.

**Methods:**

One-piece monolithic zirconia restorations, with angulated screw channels set at 0°, 15°, and 25° (*n* = 6 per angulation) were fabricated and bonded to titanium inserts using a dual-cure adhesive resin cement. These assemblies were then screwed to implant fixtures embedded in acrylic resin using an omnigrip screwdriver, and reverse torque values were recorded before and after thermomechanical cycles. Additionally, fracture modes were assessed subsequent to the application of compressive load. One-way ANOVA and Bonferroni post hoc test were used to compare the groups (α = 0.05).

**Results:**

The study groups were significantly different regarding the fracture resistance (*P* = 0.0015), but only insignificantly different in the mean percentage torque loss (*P* = 0.4400). Specifically, the fracture resistance of the 15° group was insignificantly higher compared to the 0° group (*P* = 0.9037), but significantly higher compared to the 25° group (*P* = 0.0051). Furthermore, the fracture resistance of the 0° group was significantly higher than that of the 25° group (*P* = 0.0114).

**Conclusions:**

One-piece hybrid monolithic zirconia restorations with angulated screw channels can be considered an acceptable choice for angulated implants in esthetic areas, providing satisfactory fracture strength and torque loss.

**Supplementary Information:**

The online version contains supplementary material available at 10.1186/s12903-024-04171-3.

## Background

Implant-supported prosthetics, known for their predictability and a survival rate exceeding 90%, are excellent choices for restoring edentulous areas. These restorations can be either cement-retained or screw-retained, with the latter being available in two abutment configurations: separate (two-piece) or integrated (one-piece) [1]. The straightforward prefabricated titanium abutments may not be applicable in areas such as anterior maxilla, where anatomical constraints prevent ideal implant positioning and angulation [2, 3]. Alternatively, angulation has been introduced to abutments (ranging from 15° to 30°). This alterations is directly related with the abutments’ wall thickness [4], which affects the shear force and wall strength, and therefore increase the risk of fracture. Moreover, they are prone to retaining excess cement, a notable drawback associated with peri-implant diseases [5].

**Fig. 1:**
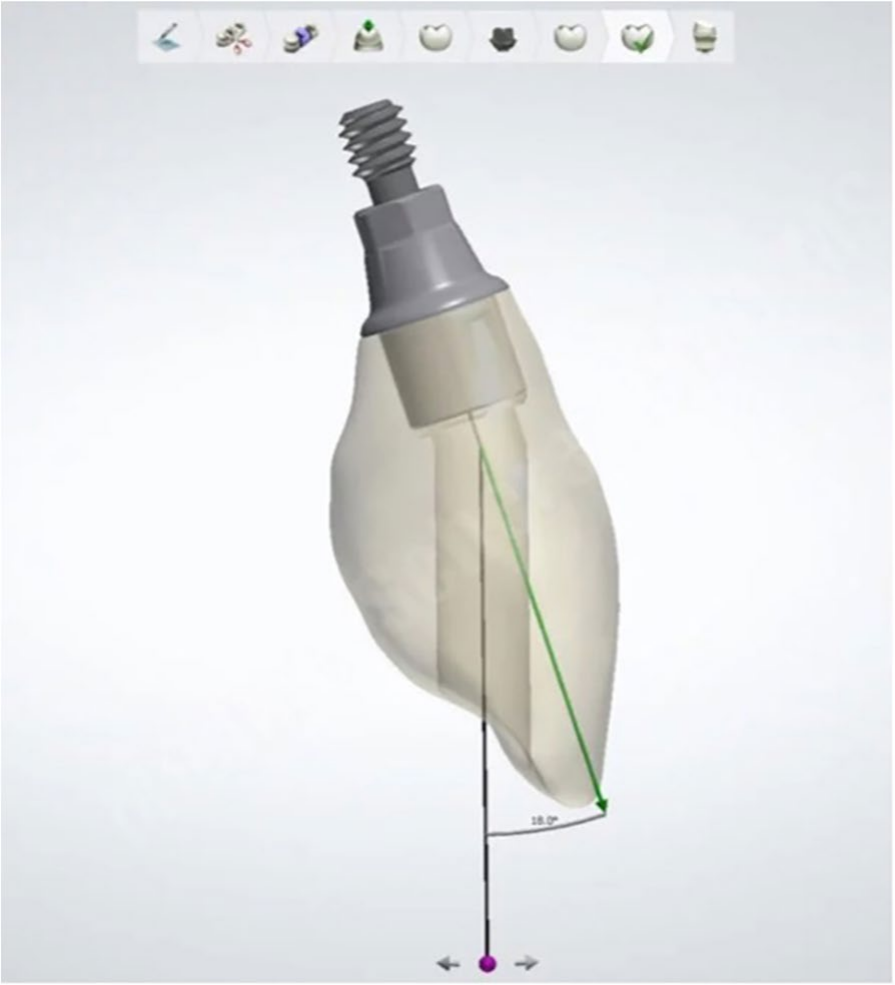
Angulated screw channel in the anterior maxilla

Screw-retained crowns (Fig. 1) are preferred for immediate loading due to the absence of residual cement, which facilitates their removal for repairs or adjustments. However, their screw access channel may affect esthetics. In cases where the residual alveolar ridge morphology in the anterior esthetic region necessitates angulated placement of the implant crown relative to the implant’s long axis, the screw access channel might emerge through the crown’s facial surface [6].

Nobel Biocare has recently introduced angulated screw channel (ASC) crowns, which allow for screw access placement at angles up to 25° from the implant axis within a 360° radius. This innovation eliminates the need for a facial access hole, thus preserving esthetics. The system employs a burnout component which resembles the screw channel access and can be adjusted to various angles on a titanium insert [7]. ASC restorations have streamlined the direct restoration of tilted implants by obviating the need for an intermediary abutment, avoiding guided bone regeneration, and allowing for a screw-retained restoration with a concealed screw channel [6].

Ceramic materials, such as zirconia, are preferred over metals for their ability to address restorations’ esthetic concerns [8–11]. Nevertheless, despite the exceptional mechanical, chemical, and esthetical properties of zirconia abutments, clinical failures have been reported [5, 9, 12]. Integrating zirconia abutment and zirconia restoration into a one-piece structure creates a secure monolithic restoration for implant attachment. A comprehensive solution that combines strength and esthetics is the hybrid abutment, whish consists of a titanium insert screwed to the implant body, paired with a superstructure made from materials such as lithium disilicate, zirconia ceramic, or resin-based composites [13–15]. The two components are assembled extra-orally either through friction fit or bonding with resin-based cement. The superstructure is fabricated by using computer-aided design-computer-aided manufacturing (CAD-CAM) or heat-pressing techniques [8, 16, 17].

Titanium base abutments have shown high fracture strength, adequate retention, and favorable marginal and internal fit in vitro, as well as comparability to conventional abutments in short-term in-vivo evaluations [3, 5, 10]. A systematic review indicated that one-piece zirconia implant restorations tend to be more fracture resistant than their two-piece counterparts. Additionally, it was advised to refrain abutment preparation for one-piece zirconia implants due to its adverse impact on fracture resistance [15].

Contradictory in-vitro findings have been reported regarding the correlation between zirconia abutments channel angulation and fracture resistance [7, 18]. A retrospective survival analysis reported that screw-retained fixed partial dentures with ASC were equally successful as those without it (94%). Potentially, ASC may receive off-axis loading, resulting in more mechanical complications than the conventional implants. This is because, theoretically, angulated torque-generating force is less effectively transferred and therefore yields reduced preload [19].

There are limited research in this area and almost no study evaluating the effect of various angulations up to 25° on fracture resistance and reverse torque value in one-piece angulated screw channel hybrid monolithic zirconia restorations after long term thermomechanical cycling simulating the long term application of these restorations in the oral cavity. Therefore, the current study was designed to address this gap and investigate how implant angulation affects the fracture resistance and reverse torque value of one-piece angulated screw channel hybrid monolithic zirconia restorations subjected to thermomechanical cycling. Additionally, this study also evaluated these restorations’ fracture mode. The null hypothesis posited that angulation would not affect the fracture resistance and mean percentage difference of reverse torque values.

## Methods

In this in-vitro experimental study, 18 implants (3.8×8 mm, TLR3809; BioHorizons Tapered internal connection, BioHorizons, USA) were embedded in chemically-activated acrylic resin blocks (Acropars Re; Marlic, Tehran, Iran) up to the implant platforms’ height to replicate bone-level implant placement. The implants’ threads were thinly coated with a dual-polymerizing composite resin (Rock Core, Zest Dental Solutions) to simulate osseointegration. The acrylic blocks were left to polymerize for 24 h.

### Sample size

According to a previous study [5] and considering a 5% significance level and power (1-β) of 90%, and sd1 = 47, sd2 = 133, mean difference (d) = 318 and one by one ratio (r) = 1 with the formula $n=\frac{\frac{1+r}{r}{s}^{2}({z}_{1-^{\alpha }\!\left/ \!_{2}\right.}+{z}_{1-\beta }{)}^{2}}{(d{)}^{2}}$, the sample size was estimated 6 per each group (total = 18).

The specimens were divided into three groups (*n* = 6 per group) based on implant angulation: 0° (following the original root configuration), 15° (moderate deviation towards the facial relative to the implant crown), and 25° (more pronounced deviation towards the facial). For angulated implants, the upper part of the implant platform was aligned with the upper part of the acrylic resin block. A dental surveyor (Marathon-103; Saeyang, Daegu, Korea) was used to ensure the precise inclination of the implant fixture to the long axis, which represented the occlusal force direction.

A wax pattern replicating a maxillary central incisor crown was fabricated, featuring a 1.5-mm contact platform parallel to the jig’s surface and a 2-mm indentation point below the incisal edge on the mesiodistal lingual surface, intended for the testing machine’s indenter. The central incisor dimensions were 11 mm in cervico-incisal length, 8.5 mm mesiodistal diameter and 7 mm labio-lingual diameter. The wax pattern was scanned (3shape D1000, 3Shape, USA, with 5-micron accuracy) to generate a standard tessellation language (STL) file, which ensures uniform size and anatomy for replicating all crowns.

The implant specimens were scanned (3Shape D1000, 3Shape, USA) using scan abutments (PYSSB8; Scan Abutment, BioHorizon, USA) with respect to the implant angulations. Subsequently, a 3D imaging software (Dental System 2022; 3shape, Denmark) was used to digitally design one-piece angulated screw channel monolithic zirconia restorations with their palatal screw access hole at 0°, 15°, and 25° angulation.

Specimens were milled (5x-300 pro; Arum Dentistry Co. Ltd, Korea) out of partially-sintered monolithic zirconia blanks (Dental Direct; Pre-shaded Zirconia Block, Advanced Material Co, Germany) and sintered in a high-temperature sintering furnace (Programat S1 1600; Ivoclar Vivadent, AG, Germany) according to the manufacturer’s instructions. Then, the fully-sintered restorations were bonded to the titanium implant inserts (PYTBL; 3.5 mm platform, GH 1 mm, 5 mm height, Laser-Lok Titanium Base Abutments, BioHorizons, USA). The bonding surfaces underwent air-abrasion with 50-µm aluminum oxide from a 10-mm distance for 10 s (0.4 MPa) according to a previous study [5]. This procedure was performed by a single skilled technician following the manufacturer’s instruction to standardize the process across all specimens. The one-piece complexes were not wholly air-abraded, given the debates about its potential impact on the flexural strength and phase transformation in yttria-stabilized zirconia.

**Fig. 2:**
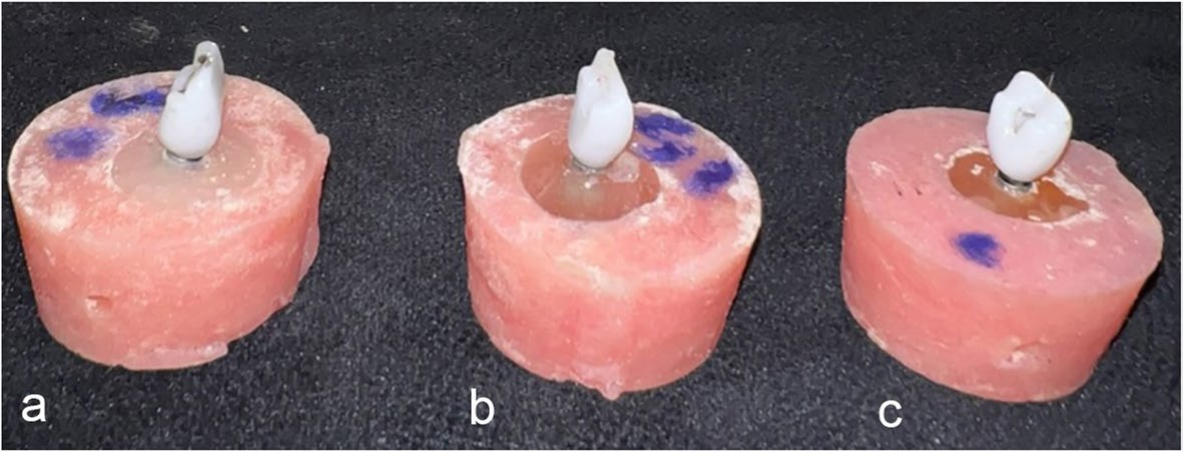
Three one-piece monolithic zirconia restorations for maxillary first incisor: **a** 0°, **b** 15°, **c** 25°

The screw holes of titanium inserts were sealed using heavy body impression material (Hydrorise Maxi Heavy; Zhermack, Germany) to prevent excessive cement from obstructing access to the screw head. Subsequently, the one-piece constructions and titanium inserts were assembled and bonded by using a dual-cure adhesive resin cement (Duo-Link Universal; Bisco, USA) following the manufacturer’s instructions. The entire assemblies were screwed to implant fixtures using titanium screws and on an omnigrip screwdriver (PADM14; Precision long driver, BioHorizons, USA), accompanied by a digital torque meter (TQ-8800; Lutron, Taiwan) to ensure torque of up to 30 N. This torque level was selected to accommodate embedment relaxation (Fig. 2). The screw was loosened after 10 min, and the reverse torque value was recorded as the initial measure (RTVi). This tightening and retightening process was repeated every 10 min for all specimens [6].

The specimens were subjected to 5000 thermal cycles between 5 ℃ and 50 ℃, with 30-second dwell times and 10-second transfer times, mirroring six months of intraoral conditions [20]. Visual inspections were conducted to detect incipient fractures and screw loosening. Any signs of ceramic cracks or fractures and screw loosening were regarded as failure.

Then, the specimens were individually mounted on a mastication simulator (CS-4.2 SD; Mechatronik; Germany) and subjected to 100 N cyclic loading at 10 Hz for one million cycles, simulating one year of functional loading [2]. The device was paused every 50,000 cycles to re-tighten any loose crowns, with each pause simulating a six-month recall appointment [2].

Following this, the specimens were visually inspected under ×10 magnification (Leica Zoom 2000; Leica Microsystems, USA) for any crown dislodgement/fracture, or screw/implant fracture. The reverse torque was remeasured and recorded as the final measure (RTVf). The two RTVs were used to calculate the percentage difference for each specimen by using the following formula [6]:

$$\mathrm{RTV}\;\mathrm{percentage}\;\mathrm{difference}=\frac{\mathrm{RTVi}-\mathrm{RTVf}}{\mathrm{RTVi}}\times100$$

**Fig. 3:**
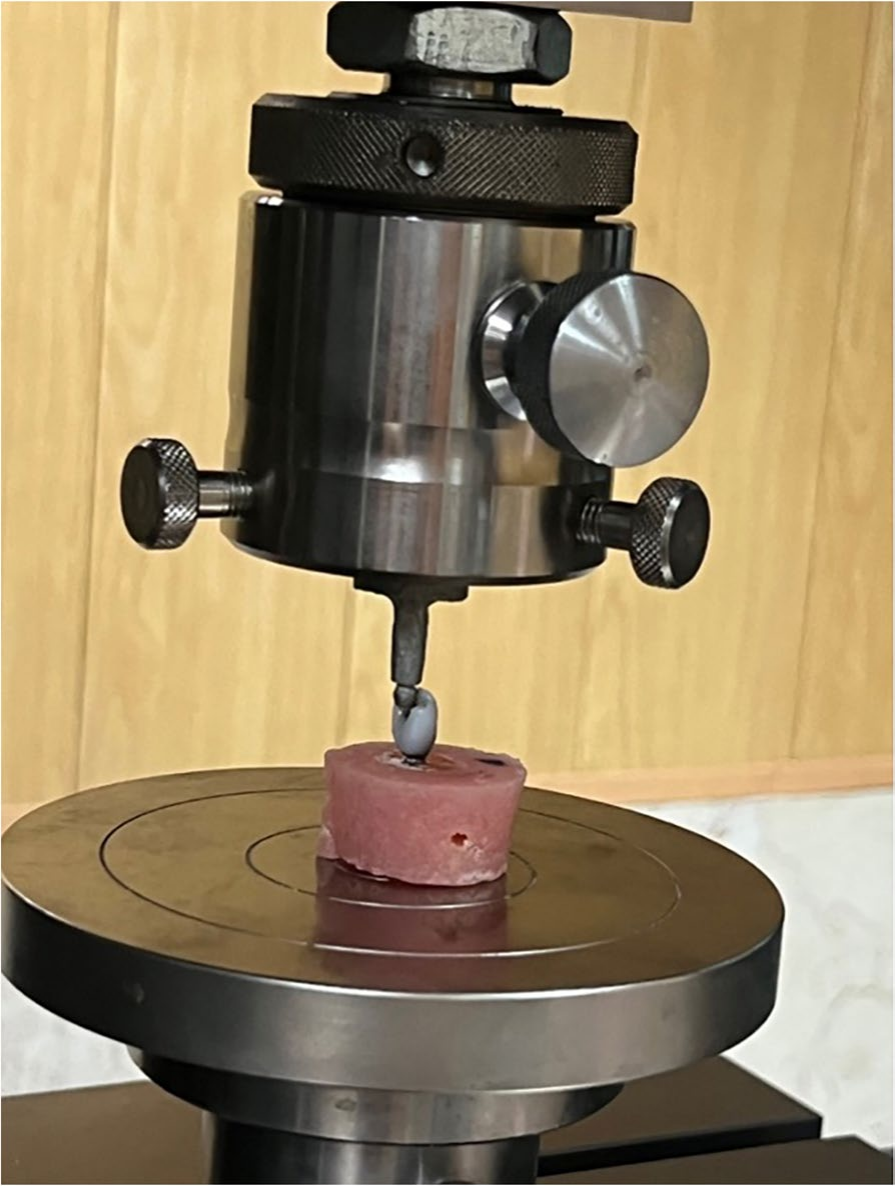
Applying compressive loading at a crosshead speed of 0.5 mm/min

The specimens underwent mechanical testing using a Universal Testing Machine (Z050; Zwick Roell, Germany), where each specimen was individually subjected to an off-axis compression load. This load was applied through contact with the machine's 4-mm mechanical indenter, positioned approximately 2 mm from the specimen's incisal edge. This setup simulated off-axis loading between the central incisor crown, supported by the implant, and the Universal Testing Machine applicator, emulating the mandibular incisor (Fig 3). To simulate the most common clinical force experienced by these teeth, a fracture test was conducted by applying a compressive load to the crown edge at a crosshead speed of 0.5 mm/min [21].

Loading was continued until a visible fracture occurred, indicated by a sudden drop in force as depicted in the graph. The force leading to fracture was recorded for each specimen, and any visible failures were documented through optical inspection. The fracture mode was evaluated based on scanning electron micrographs ([SEM], ×14 to 1000 magnifications, working distance: 9 to 13 mm, voltage: 20 kV, low-vacuum, Tescan VEGA3, Hong Kong) at ×30, ×500, and ×1000 magnifications.

The data were statistically analyzed by using SPSS software (Version 16.0; SPSS Inc., Chicago, United States). Descriptive statistics were represented as mean ± SD. Shapiro-Wilk and Levene’s tests were used to check the normal distribution of data and equality of variance, respectively. One-way ANOVA and Bonferroni post hoc test were used to compare the groups. Statistical significance was set at *P* < 0.05.

## Results

As displayed in Table 1, the data were normally distributed among the three groups since all normality values exceeded 5%. However, although the variances for mean percentage torque loss variables were equal among the three groups, there were differences in the variances for the compressive force fracture values among the three groups (*P* = 0.045).

**Table 1:** *P* values for Shapiro-Wilk and Levene’s tests

Groups		0°	15°	25°
Factors				
Normality (Shapiro-Wilk test)	Fracture resistance	0.8972	0.5242	0.2848
	Mean percentage of reverse torque	0.9662	0.1590	0.2767
Equality of variances (Levene’s test)	Compressive force fracture	0.045		
	Mean percentage of revers torque	0.151		

**Fig. 4:**
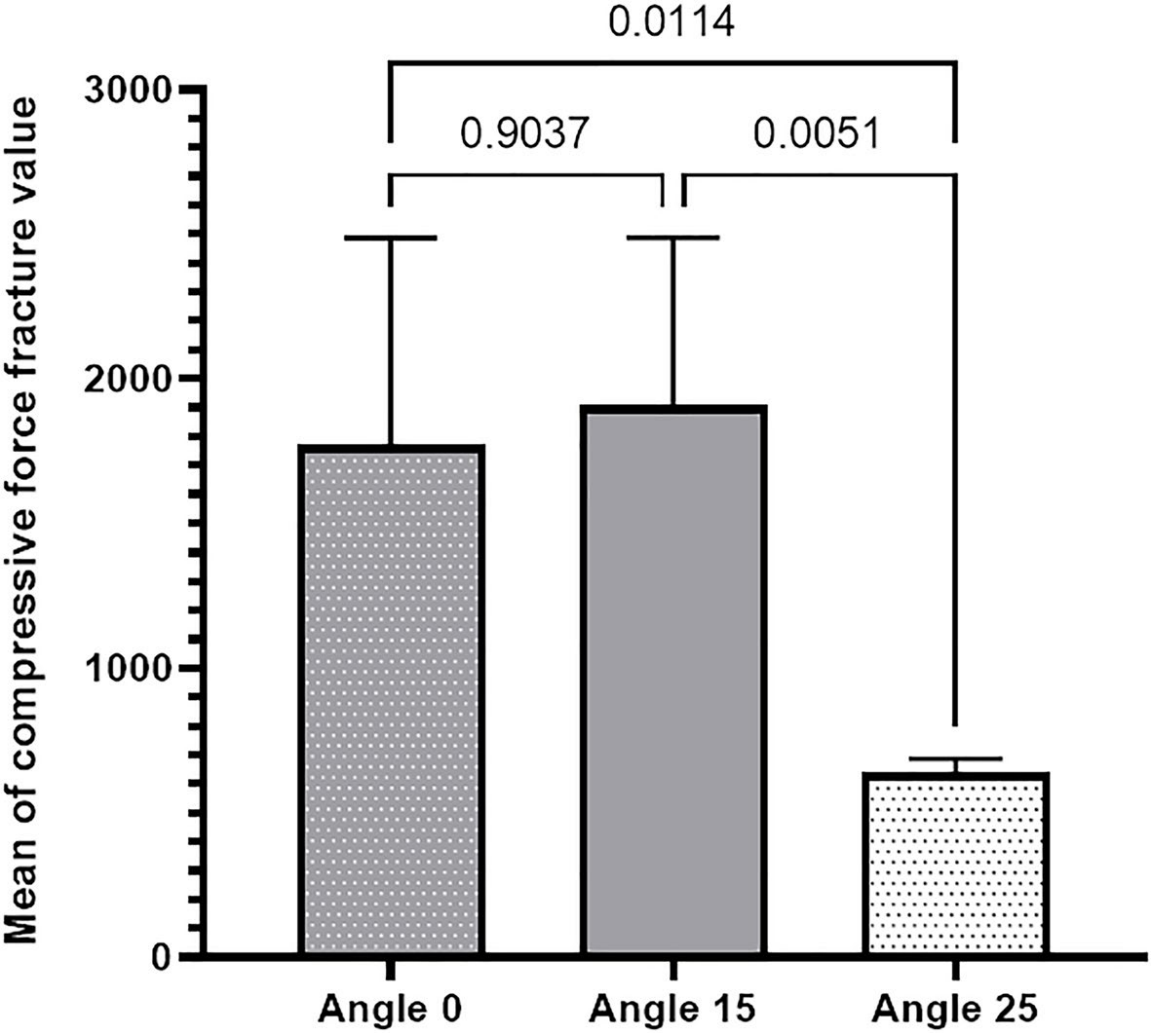
Comparing the mean fracture resistance values among the three groups

**Fig. 5:**
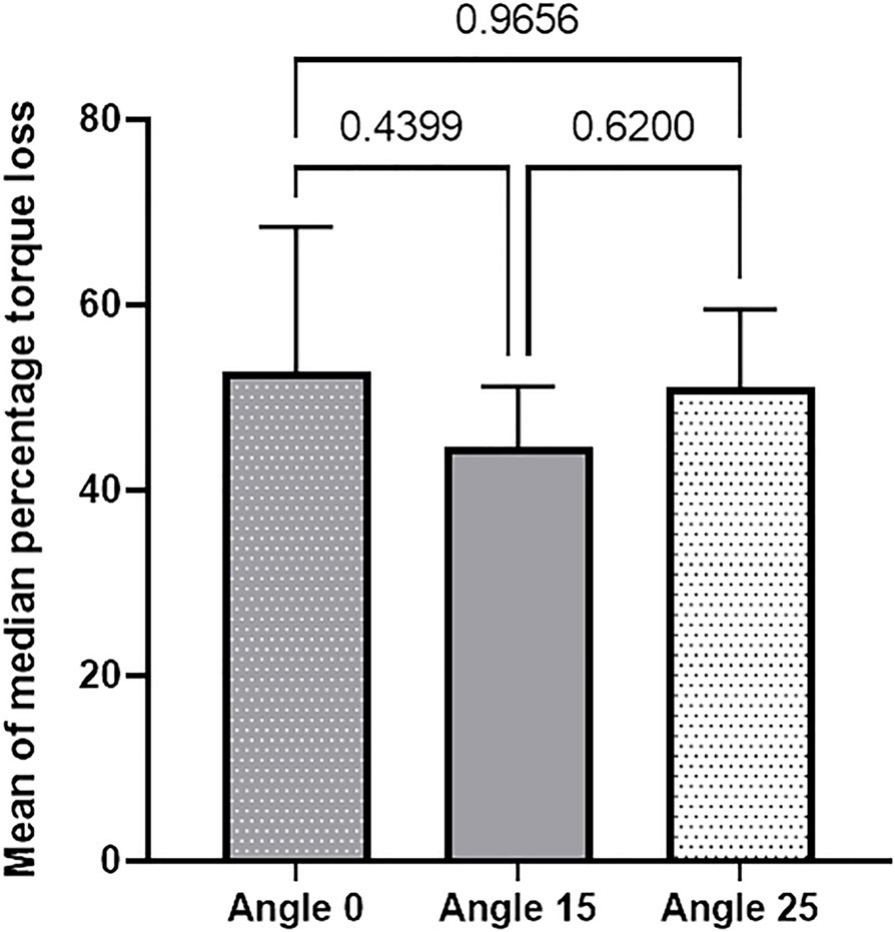
Comparing the mean percentage difference in reverse torque values among the groups

According to the results of one-way ANOVA, the differences among the three groups were statistically significant regarding the mean fracture resistance (*P* = 0.0015) (Fig. 4), but statistically insignificant regarding the mean percentage reverse torque value (*P* = 0.4400) (Fig. 5).

The highest fracture resistance was observed in the group with a 15° angulation, followed by the 0° and 25° groups. Although the difference in fracture resistance was statistically insignificant between the 15° and 0° groups (*P* = 0.9037), it was found to be statistically significant between the 0° and 25° groups (*P* = 0.0114), and between the 15° and 25° groups (*P* = 0.0051) (Table 2).

**Table 2:** Mean ± standard deviation of variables and multiple comparisons among the groups (one-way ANOVA)

Group	0°	15°	25°	*P* value
Variables				
Load at fracture (N)	1771.6 ± 714.8	1908.5 ± 580.7	638.7 ± 48.6	0.0015 ^a*^
Mean percentage reverse torque	52.7 ± 15.6	44.6 ± 6.5	51.0 ± 8.4	0.4400

^*a^Welch statistic was used due to inequality of variances

**Fig. 6:**
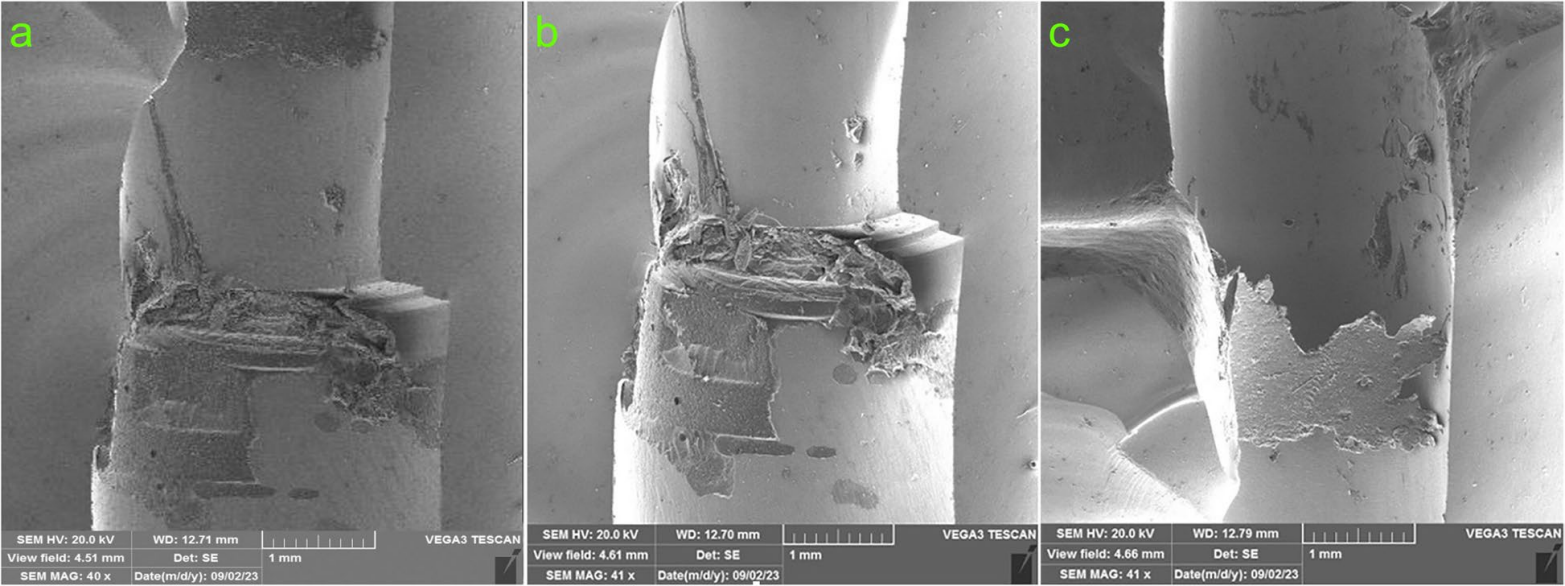
SEM overview of fractures at different groups: **a** 0°, **b** 15°, **c** 25°

**Fig. 7:**
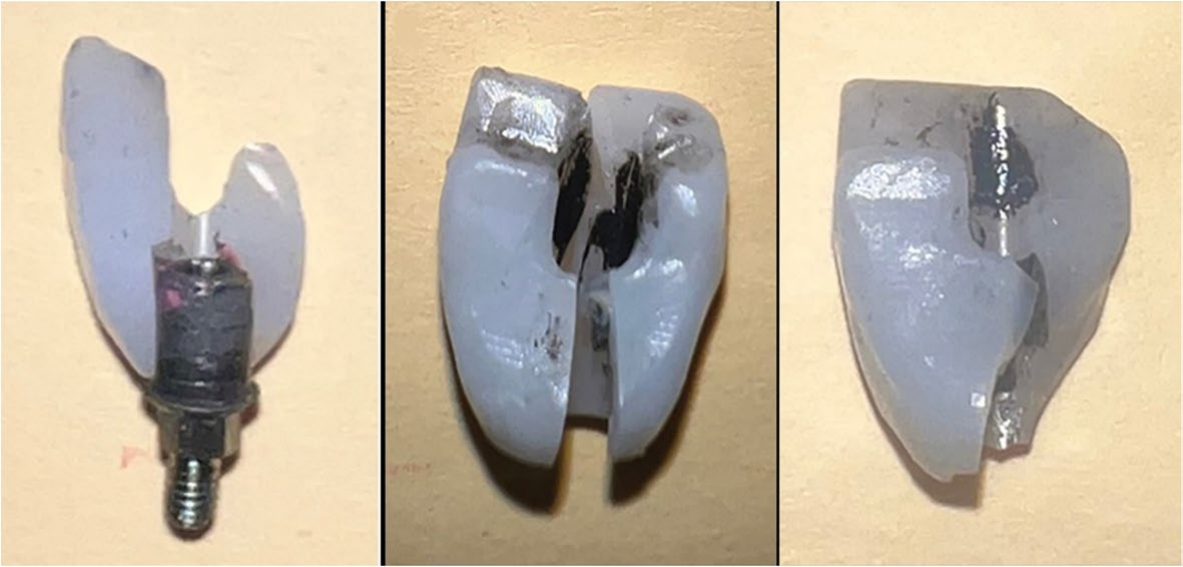
Modes of failure for all specimens in all groups (note the titanium piece of the abutment apparently intact)

The fracture test results indicated that the titanium inserts remained attached to the implant fixtures and were partially attached to the crowns in all restorations. All angulated specimens had a similar mode of fracture in the crowns, originating from the occlusal surface of restorations and extending across the screw channel hole. The partial attachment of the cement layer to the crown indicated that the resin cement layer was not the weekest point in the systems (Figs. 6 and 7).

## Discussion

The null hypothesis was partially rejected as the difference in angulation of the screw channels significantly affected fracture resistance. However, it did not significantly affect the percentage differences of reverse torque values after thermomechanical cycling.

In line with the current study, Swamidass et al. [6] evaluated the alterations in abutment screw torque in Nobel Biocare implants with straight and angled screw-access channels. They concluded that the percentage difference between RTVi and RTVf did not significantly differ between the straight and angled screw-access channel groups.

Angled abutments are occasionally recommended for non-ideal anterior implant placements. However, titanium abutments may sometimes impart an unnatural bluish appearance in the gingiva. As an alternative solution, zirconia abutments have been proposed to address this concern. Saker et al. [4] reported that straight zirconia implant abutments supporting three-unit lithium disilicate fixed dental prostheses in anterior region were more fracture resistant than those with a 15° angulation. However, this difference was not statistically significant. They also detected that the mean maximum occlusal force in the anterior region ranged from 250 N to 300 N, with some patients generating up to 605 N of force. Regardless of fatigue loading, fracture resistance in both straight and angulated specimens fell within the physiologic range, consistent with the findings of the present study.

Garcia-Hammaker et al. [7] detected that zirconia abutments with a straight channel (0°) were significantly more fracture resistant than the angulated ones (30°). They noted that for zirconia abutments with a 25° screw channel angulation, failure occurred at the apical portion of the zirconia piece within the two-piece abutment, with minor damage to the screw head. Notably, no visible plastic deformation was observed in the titanium base or implant replica, aligning with the findings of the current study.

In the present study, angulating the screw channel to 15° yielded the highest compressive strength. This result is likely due to the greater thickness of zirconia between the incisal edge and screw hole compared to the 0° group. Conversely, the 25° group exhibited the lowest fracture resistance, possibly because of the reduced circumferential ceramic material and the non-axial force.

Elsayed et al. [22] examined the fracture strength of various abutment types, including titanium, zirconia, and lithium disilicate with titanium inserts, as well as the combination of lithium disilicate abutments and crowns featuring titanium inserts. All abutments were restored with lithium disilicate crowns and attached to standard diameter titanium implants. They observed that the one-piece zirconia abutments exhibited the lowest fracture resistance, with fractures occurring at or above the implant shoulder level. In contrast, the other abutment types with titanium inserts were significantly more fracture resistant, with failure attributed to the bending of the titanium inserts and screws.

Katsavochristou et al. [18] investigated the fracture resistance and performance of zirconia implant abutments with different angulations. Similar to the present study, they discovered that an implant-to-abutment angulation of 15° resulted in a significantly higher fracture resistance compared to 0° and 25°. They also noted that, based on standard deviations, the data for the 0° group were notably more reliable. The standard deviation can be influenced by various factors including the material intrinsic properties, manufacturing factors during abutment milling, and operator’s potential errors when using the universal testing machine. Moreover, the specimens with a 25° angulation were less fracture-resistant than the straight ones (0°); which agrees with the findings of the current study.

Conversely, Albosefi et al. [23] detected that compromised implant positions requiring 15° angulated zirconia custom abutments were less fracture-resistant than the straight ones. This disparity can likely be ascribed to the reduced bulk thickness in zirconia abutments when compared to the custom monolithic zirconia restorations utilized in the current study.

Adolfi et al. [24] evaluated the impact of resin cement on torque loss, vertical misfit, and stress concentration in zirconia restorations cemented to titanium base abutments, as compared to those notched to a titanium base using the hexagon shape of the inner surface of zirconia crowns and the outer surface of the titanium base. Their findings revealed that cement-retained restorations showed a significant decrease in torque loss, stress concentration, and vertical misfit as compared to notched-retained restorations.

Goldberg et al. [25] found no significant differences in the removal torque values and fracture strength of abutment screws among the restorations with dynamic abutment angulations of 0°, 20°, and 28°, nor within the groups. However, they observed different failure patterns ranging from damaged implant platform (although the crowns remained intact) to deformed/loosened screws. This could be attributed to the fact that in that study, the specimens featured casting crowns produced on castable abutments. Apparently, all-ceramic restoration are a more favorable choice compared to metal-ceramic ones, probably due to the fracture mode that does not affect the implant and screws.

Al-Zordk et al. [26] observed that the superstructure material (zirconia, lithium disilicate, or polyetheretherketone) did not significantly affect the torque loss in titanium bases. Internal and marginal fit of titanium base abutments were comparable to other materials. The authors also found that, in cement-retained restorations with titanium base abutments, superior fit and reduced stress were observed compared to one-piece screw-retained restorations.

In the present study, the 15° group exhibited the lowest torque loss, as expected, given the torque loss associated with the increased screwdriver insertion angle. Conversely, the 0° group exhibited the highest torque loss among the three groups, possibly due to the universal screwdriver design, which features a spherical tip that becomes cylindrical as it moves downward. The asymmetrical design of the driver head might facilitate closer engagement between the screwdriver and abutment screw at 15° angulation, potentially transferring less torque to the screw at 0° and 25°. However, the difference in torque loss values in the current study was not statistically significant.

Another study, which did not involve cyclic loading reported no significant difference in RTVs between the 0° and 15° angulation groups. However, when the angulation reached or exceeded 25°, a significant reduction in RTV was observed [27]. An in-vitro study revealed that a 10° angulation yielded higher RTVs compared to 0° and 20° angles. The authors speculated that the inherent asymmetry in the driver head design facilitated closer contact between the screwdriver and the abutment screw at 10°, as opposed to the 0° and 20° angles. Hu et al. [28] reached a conclusion consistent with the present study, suggesting that 20° of angulation yielded the lowest mean RTVs, while the mean RTVs of the 10° group were the highest among the groups.

Mulla et al. [2] investigated the impact of cyclic loading on RTVs in ASC crowns with a 25° angle correction using angled titanium bases. Their findings revealed a significant difference in the RTVi between angulated specimens (25°) and straight control crowns (0°). Although angulated crowns (25°) produced lower torque values than the straight ones (0°), their resulting RTVs after cyclic loading did not exhibit significant differences, consistent with Swamidass’s study [6].

Bonyatpour et al. [5] reported that implant angulation significantly influenced the fracture resistance of one-piece screw-retained hybrid monolithic zirconia restorations. In line with the present study, they observed that 15° of angulation resulted in a significantly higher resistance than 0°. However, in contrast to the present study, they reported that 25° of angulation performed even better than 0°. The difference may stem from the type of specimens used, which were premolars in their study (versus the incisal specimens in the present one). Additionally, it is noteworthy that as the angulation decreases, the hole angle becomes larger, resulting in reduced circumferential material. They observed fractures only in the restorations, not at the screw levels, which aligns with the findings of the current study.

The findings of this study suggest that clinicians can confidently consider one-piece angulated screw channel hybrid monolithic zirconia restorations to ensure both strength and stability in the esthetic zone, thereby enhancing patient outcomes. These restorations exhibit satisfactory fracture strength and minimal torque loss, highlighting their potential clinical utility in implant dentistry.

In this in-vitro study, replicating precise oral conditions and dynamic occlusal movements proved challenging due to the nature of the applied loads (cyclic and static). Therefore, clinical research is required to validate these findings. Furthermore, future studies could explore other ceramic restoration materials and angulated titanium bases to expand upon the findings of this study.

## Conclusions

Within the limitations of this study, the following conclusions can be drawn:

1. One-piece screw-retained hybrid monolithic zirconia restorations with angulations up to 15° in screw channel are significantly more fracture-resistant than those with 25° and 0° angulations.
2. Increasing the implant-to-restoration angulation from 0° to 25° significantly reduces the fracture resistances of ASC crowns. Nevertheless, it remains within the range of clinical endurance for the mean maximum occlusal force in the anterior region.
3. Angulation up to 25° in the screw channel of monolithic zirconia restorations do not affect the reverse torque value of restorations.

### Supplementary Information

**Additional file 1.** Raw data for all study groups.

## Data Availability

All data generated or analysed during this study are included within the article and its supplementary information files.

## Abbreviations

ASC: Angulated screw channel
CAD-CAM: Computer-aided design-computer-aided manufacturing
3D: Three-dimensional
SEM: Scanning electron microscopy
RTV: Reverse torque value
